# Influence of Temperature on Void Collapse in Single Crystal Nickel under Hydrostatic Compression

**DOI:** 10.3390/ma14092369

**Published:** 2021-05-02

**Authors:** Mahesh R. G. Prasad, Anupam Neogi, Napat Vajragupta, Rebecca Janisch, Alexander Hartmaier

**Affiliations:** Interdisciplinary Centre for Advanced Materials Simulation, Ruhr-Universität Bochum, 44801 Bochum, Germany; mahesh.prasad@rub.de (M.R.G.P.); napat.vajragupta@rub.de (N.V.); rebecca.janisch@rub.de (R.J.); alexander.hartmaier@rub.de (A.H.)

**Keywords:** molecular dynamics, void, hot isostatic pressing

## Abstract

Employing atomistic simulations, we investigated the void collapse mechanisms in single crystal Ni during hydrostatic compression and explored how the atomistic mechanisms of void collapse are influenced by temperature. Our results suggest that the emission and associated mutual interactions of dislocation loops around the void is the primary mechanism of void collapse, irrespective of the temperature. The rate of void collapse is almost insensitive to the temperature, and the process is not thermally activated until a high temperature (∼1200–1500 K) is reached. Our simulations reveal that, at elevated temperatures, dislocation motion is assisted by vacancy diffusion and consequently the void is observed to collapse continuously without showing appreciable strain hardening around it. In contrast, at low and ambient temperatures (1 and 300 K), void collapse is delayed after an initial stage of closure due to significant strain hardening around the void. Furthermore, we observe that the dislocation network produced during void collapse remains the sample even after complete void collapse, as was observed in a recent experiment of nickel-base superalloy after hot isostatic pressing.

## 1. Introduction

The mechanical properties of materials are significantly influenced by the defects, generated during fabrication. Conventional manufacturing techniques like casting, forming, and moulding, and also state-of-the-art techniques like additive manufacturing (AM) produce various kind of defects, such as vacancies, dislocations, voids, and cracks, in the fabricated parts, depending on the choice of the processing parameters and routes. Amongst them, voids are commonly observed and they often exhibit detrimental effect on mechanical properties of the fabricated metallic component.

In metals and their alloys, failure occurs at the component length scale, however, it is typically a consequence of the growth and the coalescence of voids occurring at the atomistic length scale [1,2,3,4,5]. So far, it is understood from the atomistic studies that the voids facilitate dislocation nucleation at the first stage of applied loading. Consequently, in the second stage the voids grow while the dislocation loops around them glide and interact [1,2]. The voids eventually coalesce as a precursor to fracture [1]. In order to reduce the voids and eliminate their adverse effects, processes like Hot Isostatic Pressing (HIP) and forging are applied on the manufactured components [6,7]. For instance, in recent years, the high-resolution X-ray computed tomography (XCT) of Ti- [8] and Ni-based HIPed superalloys [9,10,11] confirms that HIPing is an effective way to diminish voids. In this context, Prasad et al. [12] used a crystal plasticity model to study the effect of applied pressure and holding time on the reduction of the porosity in nickel-base superalloy.

Experimental observations backed by quantitative measures allow qualitative insights into the mechanism of void growth or collapse. Observations can be made on the dislocation networks, the vacancies and the void fractions of the final state of the specimen microstructure subjected to mechanical loading. However, it is challenging to gain a comprehensive understanding of the exact deformation mechanisms that lead to the growth/collapse of the void, experimentally. In this context, a plausible and complementary approach are numerical simulations, to better understand the involved intricacies. In this regard, based on the approximation of a porous solid having a thick walled spherical shell [13], Gurson [14] proposed a phenomenological constitutive relationship, which considers the voids in terms of a single parameter of void volume fraction. This Gurson model [14] has been modified by Needleman [15] and Tvergaard [3], to take into account the effect of void size and shape on the porosity evolution and the strength reduction due to void growth. Starting from the Gurson model [14], many continuum models [16,17,18] are developed to demonstrate void growth and coalescence considering the lattice orientation and associated void geometry. While these continuum models are efficient in describing the evolution dynamics of porosity and its effect on the yield strength with close agreement with experiments, the mechanisms of void growth and coalescence, and how they depend on the void geometry and orientation, are still lacking in the models. The atomistic simulations are therefore an absolute requirement to understand the event of void growth/collapse by elementary mechanisms, such as dislocation generation, interactions and vacancy diffusion.

Previous atomistic simulations uncover that dislocation emission from the growing voids is the primary mechanism of radial material transfer required for void expansion [1,2,19,20]. MD simulations also have reported prismatic and shear loop formation, which accommodate the strain gradients around the voids. Hence, these dislocations are geometrically necessary dislocations (GNDs) [1,21,22,23]. Through MD simulations Rudd, Lubarda, and Traiviratana et al. [1,19,21] showed that voids grow by the sequential nucleation, growth and expansion of loops from the void surface. The process of the growth and coalescence of voids via emission of GNDs from grain boundaries has also been unraveled. Under high-strain rate dynamic loading, the voids at the early stage act as if they are isolated and emit dislocation shear loops, while at later stages of deformation, strain hardening is observed due to dislocation-dislocation interaction and formation of a dislocation forest [24,25,26,27,28,29].

Other MD investigations of the void growth and coalescence are addressed to specific materials, e.g., nickel [30], copper [31], magnesium [32], aluminum [33], vanadium [34], and γ-TiAl [35]. In contrast, MD studies that investigate the mechanism of void collapse can be distinguished based on the type of external loading—uniaxial compression (e.g., forging) or hydrostatic compression (e.g., HIP). The atomistic pathway of void collapse mechanisms under uniaxial loading in fcc [26,27,36] and bcc [37] metals are investigated in the previous works. Other reports, such as that of Xu et al. [38], analyzed the dependency of the specimen size, strain rate and the initial void volume fraction on the void collapse for γ-TiAl single crystals. Zhang et al. [39] investigated the influence of void density on the dislocation mechanism responsible for void shrinkage in nickel single crystals. They found, that if at constant strain, the void density is increased, and subsequently the total dislocation density is also increased and thus the material yielding accelerates.

MD studies which investigate the effect of hydrostatic compression on the void collapse mechanism are few in number. With respect to the collapse of nano-voids in tantalum single crystals under both uniaxial and hydrostatic compression, Tang et al. [40] show that dislocation shear loops are generated and they evolve with the applied loading. In addition to shear loops, prismatic loops are generated under hydrostatic compression and evolve by detaching from the void’s surface. Guan et al. [41] studied the effect of void size and initial temperatures on the nano-void collapse in aluminium single crystals subjected to both uniaxial and hydrostatic compression loading. They found that, during hydrostatic compression, the dislocation networks form tetrahedrons around the void, which reduces the void collapse rate. The work of Xu et al. [42] focuses on hcp-Ti to study the void collapse mechanism and the effect of void size on the void collapse rate subjected to hydrostatic compression. Although the aforementioned studies provide insights into the void collapse mechanism and its intricacies, an understanding of the influence of temperature on the collapse mechanism is still missing. Temperature has a significant influence on the void collapse rate due to vacancy diffusion, which was investigated experimentally for nickel-based superalloys by Epishin et al. [9,10,43].

The goal of the current investigation is, thus, to employ MD simulations to gain a deeper understanding of the mechanism of void collapse under hydrostatic compression in single crystal nickel at various temperatures. Emphasis is placed on the effect of temperature on the formation of dislocation networks and vacancies responsible for the void collapse. Void volume fraction, density of nickel atoms in the void region, the mean square displacement (MSD) and the radial distribution function (RDF) are used to analyze and quantify various aspects of the void collapse region. Section 2 details the MD model along with the simulation parameters used in the current work. In Section 3, first, the mechanical behavior in the form of stress-strain curves is calculated to demonstrate the effect of temperature, and other important characteristics describing the void collapse are also discussed here in detail. Next, owing to the significant influence of the dislocations on the void collapse, the mechanisms of dislocation nucleation, evolution, and interactions are detailed with the help of MD simulation snapshots. The void collapse mechanisms at temperatures 1 K and 1500 K are described in Section 3.4 and Section 3.5. Section 4 summarizes the results of the current work and presents conclusions.

## 2. Simulation Method

Molecular dynamics simulations are performed using the Large-scale Atomic/Molecular Massively Parallel Simulator, LAMMPS [44]. We first generate a single crystal in such a way that the *X*, *Y* and *Z* orthogonal directions are oriented along [100], [010], and [001] directions. The atomic interactions of the considered nickel atoms are modeled by using the embedded atom method (EAM) potential developed by Mishin et al. [45]. It is worth noting that this Mishin EAM potential for Ni [45] was developed by fitting to experimental and as well as first principle data, and it can reproduce lattice properties, point defects and planar faults of Ni with considerable accuracy as compared with experiments. Therefore, considering the predictive accuracy and ability to describe high temperature deformation [46,47], Mishin-EAM potential is chosen to be employed in the current simulations performed within this study.

**Figure 1:**
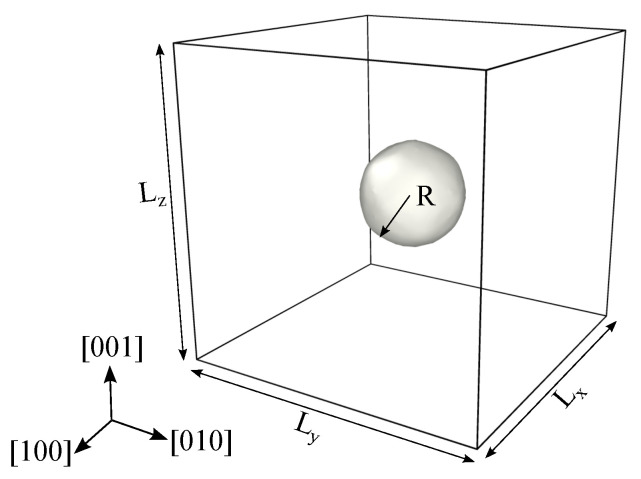
Schematics of the simulation setup with $L_{x},\;L_{y}$ and $L_{z}$ representing the specimen side dimensions along [100], [010] and [001] directions. *R* represents the radius of the void, and periodic boundary conditions are enforced on all three directions. Hydrostatic compressive load is applied on the six faces of the simulation box.

The initial bulk single crystal Ni sample is equilibrated by using the isothermal isobaric NPT ensemble at ambient temperature and pressure, using time-step of 1 fs. Periodic boundary condition is maintained throughout all the simulations performed in this study unless mentioned otherwise. After successful equilibration, a spherical void is introduced at the center of the bulk sample by eliminating Ni atoms. The sample with the void is then re-thermalized by employing the isothermal isobaric NPT ensemble at ambient temperature and pressure. As the objective of this work is to unravel the effect of the initial temperature on deformation, the NPT ensemble is employed further for 100 ps to prepare samples with different initial temperatures varying from 1 K to 1500 K with an increment of 300 K. The initial simulation set-up is presented schematically in Figure 1. To eliminate any finite-size effect on the void collapse mechanism, additional simulations are performed up to a cell dimension of 28.16 nm and a void size of 4.694 nm to study the influence of the sample and void size on the void collapse mechanism. According to this study, it was found that a cubic simulation cell with a dimension of 14.1 nm and a void radius of ∼2.3 nm is adequate to avoid any size effect. In accordance with this size effect study, simulation parameters are chosen as summarized in Table 1.

The equilibrated samples with different initial temperatures are then subjected to equi-triaxial hydrostatic compression by employing isothermal isobaric, NPT ensemble. From our additional set of simulations, we confirm that complete void collapse occurs at a hydrostatic pressure of ∼40 GPa. Hence, during this equi-triaxial loading, an isotropic pressure of 40 GPa is applied while the temperature is held constant at the initial temperature of the samples. In order to ensure sufficient energy conservation during loading, all simulations are performed up to 1 ns with a time-step size of 1 fs.

To identify the atomic-level defects produced during loading, various techniques such as adaptive common neighbor analysis (CNA) [48], centro-symmetry parameter (CSP) estimation, coordination per atom, have been utilized in this work. Dislocation extraction algorithm (DXA) (as implemented in program OVITO, developed by Stukowski et al. [49,50]) has been employed. DXA represents the dislocated crystal into a line-based form of the dislocation network. Apart from distinguishing between different dislocation mechanisms, DXA is capable of recognizing the true Burgers vector of each dislocation segment [51,52], grain boundary dislocations (e.g., twining dislocation), and also dislocation junctions [53]. The Voronoi tessellation analysis technique is used to calculate the atomic volume to quantify the void volume fraction. We use Wigner-Seitz defect analysis to capture vacancy formation during loading.

## 3. Results and Discussion

### 3.1. Void Closure Strains

**Figure 2:**
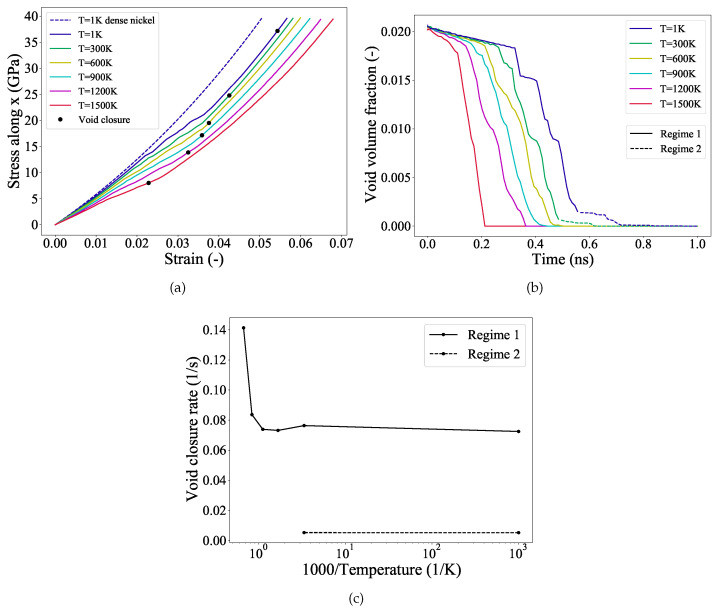
Effect of temperature on the void collapse of single crystal nickle under hydrostatic compression. (**a**) Stress-strain curves along *x* direction plotted for various temperatures (T). The stress-strain curve forr dense Ni single crystal is plotted for comparison using a dashed line. Markers indicate the strain at which the complete closure of the void occurs. (**b**) Void volume fraction is plotted as a function of time for various temperatures (T). At 1 and 300 K, the initial stage of void collapse (regime-1), and the second stage (regime-2) are indicated respectively by solid and dashed line. (**c**) The void closure rate is plotted as a function of inverse temperatures. The solid and dashed lines represent regime-1 and regime-2.

The stress-strain curves of the samples at different temperatures are compared in Figure 2a, and the corresponding complete void closure strains are marked in the plots. For the sake of comparison between the samples with and without a void, a reference stress-strain curve for a dense nickel system is also plotted in Figure 2a. The elastic modulus and associated yield strength are observed to decrease gradually with increasing temperature. The lower the temperature the higher the yield strength. This temperature-dependent elastic modulus and yield strength indicate significant thermal softening; the sample becomes more pliable at a high temperature due to the increased kinetic energy present in the system allowing atom debonding at lower stress values. Consequently, the defect density, produced during loading, is also observed to increase with increasing temperature. Interestingly, it can also be observed from the markers of Figure 2a that the strain to complete void closure critically depends on the operating temperature; the lower the temperature the higher the strain required to close the void. For instance, void is observed to be fully closed at a strain value of ∼5.5% at 1 K while at 1500 K void is closed at a much lower applied strain of ∼2.4%.

In order to inspect the incipient pathway of void closure, the evolution of void volume fraction with time is plotted in Figure 2b. The void volume fraction is calculated using OVITO’s construct surface mesh algorith [54]. As depicted in Figure 2b, at any temperature, initially the void volume fraction decreases gradually with increasing applied load (a latency phase during elastic deformation), then at a certain critical strain value the void starts collapsing rapidly. These critical strains indicate the onset of yielding which is mediated via nucleation of dislocations around the void. It can also be observed that with increasing temperature not only the dislocations nucleate earlier (lower yield strain), but also the time to complete the closure of the void decreases. However, irrespective of the temperature, at the post-yield regime, the rate of void collapse remains almost constant. In case of low temperatures (1 and 300 K), the closure of the void is delayed after a certain stage of applied loading. For the sake of our analysis, we denote the initial stage of void closure at the post-yield regime as regime-1, as shown by the solid lines in Figure 2b. The regime-2 is shown by using the dashed lines in Figure 2b. Interestingly, at higher temperatures (600–1500 K), this regime-2 is absent and the void is observed to close continuously.

To investigate the rate of void closure at different temperatures, we have plotted the void closure rate as a function of inverse temperature in Figure 2c. The rate of void closure at regime-2 (1 and 300 K) is denoted by the dashed line. As can be observed that the rate of void closure in regime-1 is almost constant until 900 K. However, at elevated temperatures (1200–1500 K), the rate of void closure is increased by ∼72%. The rate of void closure is therefore not too sensitive to temperature, albeit this process can get thermally activated when the operating temperatures is at or above 1200 K. Furthermore, at 1 and 300 K, in the regime-2, the void collapse is delayed significantly and the rate is close to zero, Figure 2c. Therefore, it is now evident that there is a clear difference in the mechanisms of void collapse in regime- 1 and 2. We have analyzed the underlying atomistic events during the void collapse. This will be discussed in Section 3.4.

### 3.2. Diffusion of Nickel

**Figure 3:**
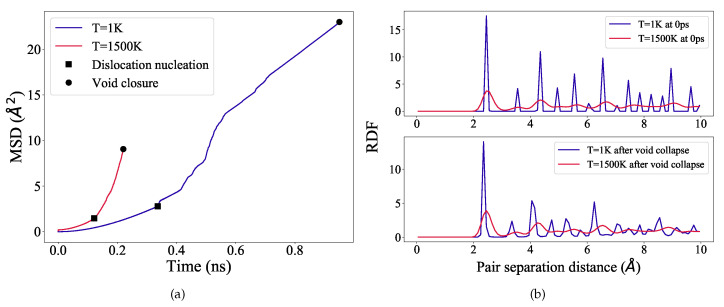
(**a**) MSD for the entire system is plotted as a function of simulation time for the temperatures 1 K and 1500 K. Markers indicate the dislocation nucleation and the complete void closure stages of the simulation. (**b**) RDF for the entire system is plotted at the start of the simulation (top) and at void collapse stage (bottom), for the temperatures 1 K and 1500 K.

To accurately examine and quantify the time-dependent diffusion characteristics, we compute mean square displacement (MSD) of the Ni atoms both at 1 K and 1500 K, Figure 3a. The markers in Figure 3a indicate the onset of dislocation nucleation (yielding) and the complete void collapse at 1 K and 1500 K.

The slope of a MSD curve represents the averaged diffusion coefficient (D) of the atoms. The MSD curve at 1500 K is relatively stiffer than at 1 K (Figure 3a), and as calculated, $D=8.3\times 10^{-11}\;\;m^{2}/s$, and $12.21\times 10^{-11}\;\;m^{2}/s$, respectively at 1 K and 1500 K. The diffusion coefficient at 1500 K is therefore substantially higher (∼1.5 times) than at 1 K. This enhanced diffusion coefficient at 1500 K clearly suggests that during deformation at higher temperatures, the sample is more susceptible to vacancy production than dislocation based plasticity.

In order to inspect the local atomistic structure of the sample after void collapse at 1 K and 1500 K, we calculate the radial distribution function (RDF), which provides the probability of finding an atom a particular distance away from another atom. The RDF is plotted in Figure 3b. As evident from Figure 3b, at 1 K, after complete void collapse the coordination peaks are somewhat broadened, however, they still remain equally intense and sharp with a long-range order up to 10 Å. At 1500 K, initially at 0 ps (before loading), the peaks exhibit significant thermal broadening with lower intensity, but the peak positions are not shifted as compared with 1 K. The peak characteristics are notably not changed even after complete void collapse. This type of low-intense and broadened peaks typically indicate a solid but pre-melting phase in the sample. It is therefore evidenced that even after high temperature loading, the sample is still solid but partially disordered. The atomic disorder is because of thermally-assisted vacancy formation and subsequent migration. However, to investigate further the local lattice structure and associated defect production during loading, we analyze our deformed samples using adaptive CNA and CSP method. The results will be discussed in the following.

### 3.3. Evolution of Defects

**Figure 4:**
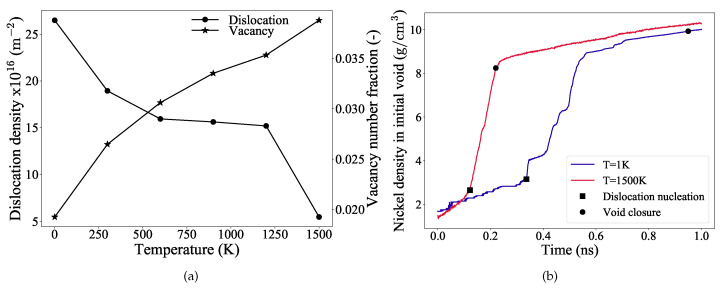
(**a**) The dislocation densities and the vacancy number fractions evaluated at the strain of complete closure of the void are plotted as a function of temperature. The data points are simply connected with solid black line as a guide for an eye. (**b**) Density of nickel atoms in the initial void region calculated as a function of simulation time for the temperatures 1 K and 1500 K. The markers indicate the dislocation nucleation and the complete void closure stages of the simulation.

To further understand the influence of dislocations and vacancies on the void collapse, the dislocation densities and the vacancy number fractions are evaluated at the void closure strains (indicated by markers in Figure 2a) and plotted as a function of temperature in Figure 4a. To characterize vacancies, we use CSP filter to distinguish defected and regular fcc atoms.

It is observed from Figure 4a that, as the temperature increases, the dislocation density decreases and correspondingly the vacancy number fraction increases. This implies that, at higher temperatures (close to 1500 K) vacancies dominate and they accelerate the void collapse mechanism. At low temperatures, dislocation based plastic deformation still predominates and contributes more to the void collapse. However, it is important to note here that these values are evaluated at the strain where complete void closure occurs. Therefore, to further investigate their individual influences, and for the sake of comparison between the highest and the lowest temperature considered in this study, more emphasis will now be placed on the results at 1 K and 1500 K.

The density of nickel atoms in the initial void region, when examined as a function of simulation time, provides insight into the diffusion characteristics of Ni atoms. Figure 4b depicts the nickel atom density inside the initial void, plotted against time for temperatures 1 K and 1500 K. The markers in this figure indicate the dislocation nucleation and the void collapse stages of the simulation. Two distinct regions are observed for both curves, although the slopes in these regions vary significantly. For the low temperature (1 K) curve, in the elastic deformation regime, the nickel atoms diffuse uniformly into the void (indicated by a nearly constant slope until dislocation nucleation). The initial fluctuations of the slope in the elastic regime is due to the smaller size of the void (R = 23 Å) considered for the simulation, Table 1. Once the plastic deformation sets in, a rapid rise in the slope occurs indicating that the dislocations mediate the increased diffusion of nickel into the void. The curve at this stage shows regions of plateau and rapid rise, which is attributed to the strengthening mechanism and is explained in detail later in the Section 3.4. As mentioned earlier, thermal softening is significant at high temperatures such as at 1500 K, which can be inferred from Figure 3a,b also.

It is evident from Figure 4a, that the dislocations are the primary reason for the void collapse at lower temperatures. Similarly at higher temperatures, although the contribution of vacancies is significant, it is the dislocation nucleation and its evolution that accelerates the rapid collapse of the void. To better understand the exact mechanism of nucleation, evolution, and interaction of the dislocations, the simulation performed at 1K temperature is used as reference in Section 3.4. The following Section 3.4 and Section 3.5 detail the mechanism of void collapse at 1 K and 1500 K, respectively.

### 3.4. Analysis of Void Collapse at 1 K

**Figure 5:**
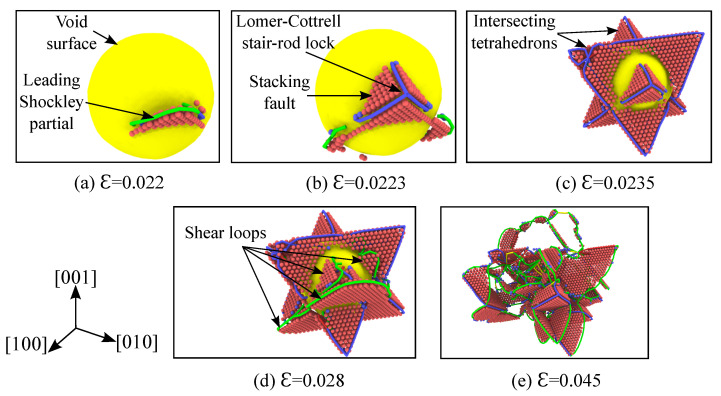
Nucleation and evolution of dislocations from the void’s outer surface at various strains for single crystal nickle under hydrostatic compression. Simulation temperature is 1K, specimen and void size are as listed in Table 1. Atoms of fcc crystal structure are hidden based on CNA analysis and the void surface is depicted with the color yellow. Based on the DXA analysis, the Shockley partials are colored green, the Lomer–Cottrell dislocations are colored blue, perfect dislocations are colored yellow and the complex dislocations are colored red. Using the centro-symmetry parameter (CSP) analysis, only the atoms belonging to the stacking faults are visualized in red color.

**Figure 6:**
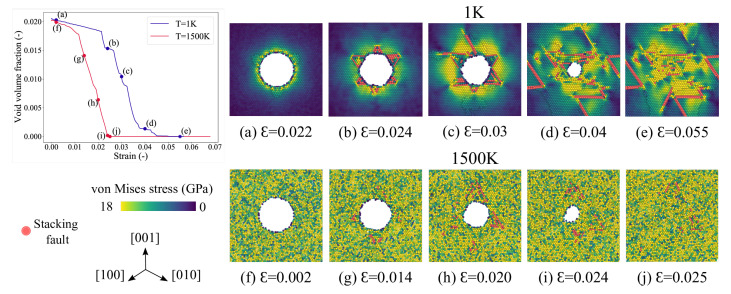
The void volume fraction vs. strain plot at T=1 K and 1500 K. Atomistic snapshots at different strain values depicting the von-Mises stress distribution at T=1 K, (**a**–**e**); and T=1500 K, (**f**–**j**). The snapshots are taken on the (111) cross-sectional planes of finite width. The atoms are colored based on their von-Mises stress values, and the atoms corresponding to stacking fault are colored red.

Figure 5a–e illustrate the nucleation and evolution of dislocations from the void’s surface undergoing hydrostatic compression at a temperature of 1 K. The corresponding stress-strain curve for this loading is depicted in Figure 2a, and the stress contours across the (111) plane at various stages of compression are shown in Figure 6.

Dislocations nucleate at the surface of the void corresponding to a volumetric strain of **ϵ=0.022**, as depicted in the Figure 5a. The shear loop consists of two layers of atoms, which represent the Shockley partial dislocations separated by a stacking fault. In fcc, both the leading and trailing partial dislocations are approximately bowed into semi-circles, which are energetically favorable dislocation configurations due to the minimum stress required [55]. As the load is further increased, more shear loops on the various close-packed {111} planes are emitted from the void’s surface. When two such evolving shear loops meet along the line of intersection, they result in the formation of a Lomer–Cottrell stair-rod lock [55]. The formation of such a lock is depicted in Figure 5b and an example reaction is given by: $\frac{a}{6}\left[\bar{1}2\bar{1}\right]+\frac{a}{6}\left[1\bar{1}2\right]\to \frac{a}{6}\left[011\right]$. Interestingly, this type of octahedral slip around the void has also been observed in the recent HIP experiment of Ni-based superalloy by Epishin et al. [43]. These stair-rod dislocations form two intersecting tetrahedrons around the void as the strain reaches a value of ϵ=0.0235, which is depicted in Figure 5c.

Since the Burgers vector of the Lomer–Cottrell dislocation is perpendicular to the line of intersection, it results in the dislocation becoming immobile. According to Frank’s rule [55], it exerts a repulsive force on the two Shockley partials and prevents the shear loops from crossing each other. This resistance offered by the sessile Lomer–Cottrell dislocation to further plastic deformation results in the strengthening behavior. As the applied load increases, two further mechanisms are observed. Firstly, more shear loops are nucleated from the unenclosed surface of the void (Figure 5d). This is due to the fact that the void surface surrounded by the tetrahedrons can not emit dislocation loops anymore. Next, the Lomer–Cottrell dislocation formed at the edges of the tetrahedron extends its dislocation line to accommodate the two evolving shear loops. During this evolution, the trailing Shockley partial follows the leading one and releases the Lomer–Cottrell lock (Figure 5e). As these dislocation loops evolve, more and more Shockley partials, stacking faults, Lomer–Cottrell locks and other complex reactions occur. Production of this kind of stacking fault tetrahedron (SFT) is often observed in fcc metals under external irradiation [56] or during high-strain rate loading [57].

The evolution of the void volume fraction as a function of time is depicted in Figure 2b. Before the yield point is reached (elastic region), the void volume fraction decreases linearly with a very small slope. Once the plastic deformation begins, i.e., the shear loops start nucleating on the void’s surface, the void starts to collapse rapidly. At a strain of ϵ=0.0235, two intersecting tetrahedrons form around the void and prevent shear loops from propagating further. This strengthening mechanism described earlier with respect to the Lomer–Cottrell stair-rod lock is the reason for the observed slow collapse of the void (regime-2 in Figure 2b). The void starts to collapse rapidly again, once a critical value of strain ϵ=0.028 is reached due to the applied load. At this strain, subsequent shear loops are emitted from the unenclosed surface of the void and they cause the void to collapse rapidly again. With further loading, few more stages of strengthening and rapid collapse are observed, until the void closes completely at ϵ=0.055.

### 3.5. Analysis of Void Collapse at 1500 K

**Figure 7:**
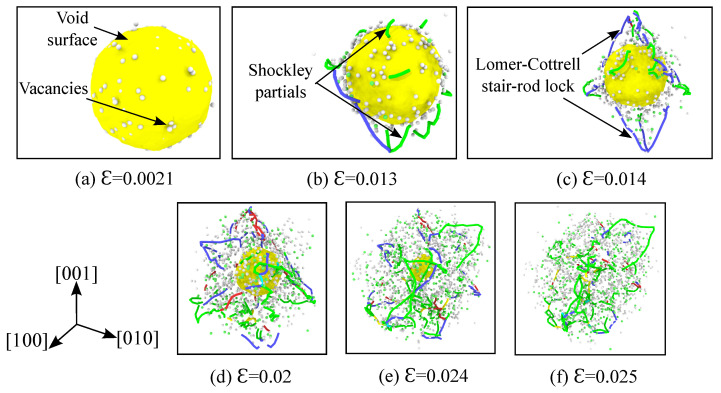
Vacancy generation, nucleation and evolution of dislocations from the void’s outer surface at various strains for single crystal nickle under hydrostatic compression. Simulation temperature is 1500 K, specimen and void size are as listed in Table 1. Atoms of the fcc crystal structure are hidden according to CNA analysis, and the void surface is depicted in yellow. Based on the DXA analysis, the Shockley partials are colored green, the Lomer–Cottrell dislocations are colored blue, perfect dislocations are colored yellow, and the complex dislocations are colored red. Using the centro-symmetry parameter (CSP) analysis, only the disordered atoms (representing vacancies) are visualized in white color.

At higher temperatures, in addition to the dislocation networks, the transport of vacancies away from the void’s surface also contributes to the collapse of the void. This is evident from Figure 4a, wherein high vacancy number fractions are observed for temperatures close to 1500 K. Figure 7a–f illustrates the void collapse, the vacancies, and the nucleation and evolution of dislocations for the 1500 K temperature simulation. The stress-strain curve for this simulation run is depicted in Figure 2a.

The mechanism of plastic deformation due to dislocation nucleation and evolution from the void’s surface observed here is similar to the one explained earlier in the Section 3.4. The void volume fraction evolution as a function of time is depicted in Figure 2b. In the elastic region the void volume fraction decreases linearly, but the slope of this region is higher in comparison to that at 1 K. This is due to the migration of vacancies away from the surface of the void that is assisted by the high temperature. Figure 7a depicts the void surface and the vacancies around it at a strain of ϵ=0.0021. Since the yield strength of the material is reduced due to the high temperature, the dislocations nucleate earlier from the void’s surface and cause the rapid collapse of the void. This is shown in Figure 7b at a strain of ϵ=0.013, where the Shockley partials are seen nucleating from the void’s surface. With further loading, the Lomer–Cottrell dislocations evolve and form locks (Figure 7c,d). As explained in Section 3.4, these stair rod locks offer resistance to further plastic deformation. Although the slow collapse of the void (indicated by a plateau region for the 1 K curve) is not evident in the Figure 2b for the current temperature of 1500 K, the presence of partial intersecting tetrahedrons can be observed in the Figure 7c,d. The void closes completely at a strain of ϵ=0.025.

### 3.6. Void Closure Mechanisms

The evolution of void volume fraction as a function of strain and the associated stress contours across the (111) plane at various stages of compression for temperatures 1 K and 1500 K are shown in Figure 6. The atoms in this figure are colored according to the von-Mises stress values, and the atoms belonging to the stacking fault are colored red. By comparing the 1 K and the 1500 K figures, it is evident that before plastic deformation, the stress is concentrated around the void for the system at 1 K, whereas for the system at 1500 K, it is uniformly distributed throughout. For the 1500 K case, it can be observed that after the strain ϵ=0.020 no more dislocations are emitted from the void’s surface as the tetrahedrons completely surround it. And after the complete closure, a dislocation source (in the vicinity of the collapse) as observed for 1 K collapse at ϵ=0.055) is not observed here.

**Figure 8:**
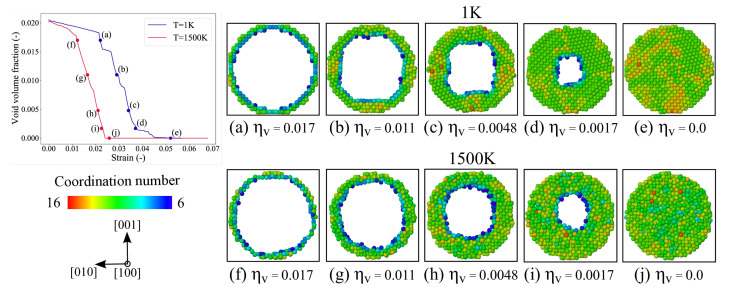
The void volume fraction vs. strain plot at T=1 K and 1500 K. Atomistic snapshots at different stages of void collapse depicting the (100) cross-sectional view at T=1 K, (**a**–**e**); and T=1500 K, (**f**–**j**). The snapshots are taken at the same volume fraction, to see the differences in pore shape. The atoms are colored based on their coordination number values.

The void closure at various stages of compression for temperatures 1 K and 1500 K is shown in Figure 8 by taking (100) plane cross-sections of finite width. The atoms in this figure are colored based on their coordination number values. It is evident from this figure that the inward collapse of the void at 1 K does not take place uniformly in all directions, but it rather begins along the principle directions of loading and then continues along other directions. The dislocation loops forming around the void transport the atoms inwards. With continued loading, this non-uniform collapse becomes more obvious. On the other hand, for the 1500 K case, although the temperature is high, the coordination numbers indicate that the material is still in the solid state. And, in contrast to the void collapse at 1 K, a uniform inward collapse from all directions is observed here. This is due to the fact that no dislocations are nucleated from the void’s surface after a strain ϵ=0.020 that directly interacts with the void.

## 4. Conclusions

In conclusion, we performed molecular dynamics simulations to understand the effect of temperature on the void collapse mechanisms in single crystal Ni under hydrostatic compression. Our simulations reveal that the void collapse mechanism is primarily mediated via nucleation of dislocations and their associated interactions, irrespective of temperature. The rate of void collapse remains temperature insensitive up to T=900 K. At or above T=1200 K, the process of void collapse becomes thermally activated and the rate of void collapse is higher compared to low temperatures.

These results indicate a change in mechanism, as at higher temperatures vacancy diffusion assists dislocation mobility and facilitate continuous void collapse, while at lower temperatures mass transport and void collapse occur via formation, glide, and interaction of dislocations, leading to strain hardening. Due to the strain hardening around the void at lower temperatures, the void collapse is delayed after an initial stage of closure compared with higher temperatures. Furthermore, the dislocation network produced during void collapse remains in the deformed sample even after complete void collapse. This is in good agreement with the reported experimental investigations of Epishin et al. [9,43] on single crystal nickel-based superalloys.

Our study offers new atomic level understanding of void collapse in Ni, and how it is influenced by the operating temperature. The observation of the dislocation network even after complete void collapse suggests that even though HIP is an effective way to eliminate voids in the fabricated metallic parts, the residual undesired defects in the HIPed microstructure could lead to poor mechanical properties and other effects like recrystallisation.

## Figures and Tables

**Table 1:** Simulation parameters considered for the temperature study. ‘a’ represents the lattice parameter of nickel (3.52 Å).

Parameters	Values
Temperature (K)	1, 300, 600, 900, 1200, 1500
Specimen size, $L_{x}=L_{y}=L_{z}$ (Å)	141
Void radius, R (Å)	23
Void volume fraction, $\eta_{v}$	0.02
Number of atoms	251,079

## Data Availability

The data presented in this study are available on request from the corresponding author.

## References

[1] Traiviratana S., Bringa E.M., Benson D.J., Meyers M.A. (2008). Void growth in metals: Atomistic calculations. Acta Mater..

[2] Bringa E.M., Traiviratana S., Meyers M.A. (2010). Void initiation in fcc metals: Effect of loading orientation and nanocrystalline effects. Acta Mater..

[3] Tvergaard V. (1989). Material failure by void growth to coalescence. Advances in Applied Mechanics.

[4] Cuitino A., Ortiz M. (1996). Ductile fracture by vacancy condensation in fcc single crystals. Acta Mater..

[5] Faleskog J., Shih C.F. (1997). Micromechanics of coalescence—I. Synergistic effects of elasticity, plastic yielding and multi-size-scale voids. J. Mech. Phys. Solids.

[6] Kellett B., Lange F.F. (1988). Experiments on pore closure during hot isostatic pressing and forging. J. Am. Ceram. Soc..

[7] Saby M., Bouchard P.O., Bernacki M. (2015). A geometry-dependent model for void closure in hot metal forming. Finite Elem. Anal. Des..

[8] Tammas-Williams S., Withers P.J., Todd I., Prangnell P.B. (2016). The effectiveness of hot isostatic pressing for closing porosity in titanium parts manufactured by selective electron beam melting. Metall. Mater. Trans. A.

[9] Epishin A., Fedelich B., Link T., Feldmann T., Svetlov I.L. (2013). Pore annihilation in a single-crystal nickel-base superalloy during hot isostatic pressing: Experiment and modelling. Mater. Sci. Eng. A.

[10] Epishin A.I., Link T., Fedelich B., Svetlov I.L., Golubovskiy E.R. (2014). Hot isostatic pressing of single-crystal nickel-base superalloys: Mechanism of pore closure and effect on Mechanical properties. MATEC Web Conf..

[11] Epishin A.I., Bokstein B.S., Svetlov I.L., Fedelich B., Feldmann T., Le Bouar Y., Ruffini A., Finel A., Viguier B., Poquillon D. (2018). A Vacancy Model of Pore Annihilation During Hot Isostatic Pressing of Single Crystals of Nickel-Base Superalloys. Inorg. Mater. Appl. Res..

[12] Prasad M.R., Gao S., Vajragupta N., Hartmaier A. (2020). Influence of Trapped Gas on Pore Healing under Hot Isostatic Pressing in Nickel-Base Superalloys. Crystals.

[13] McClintock F.A. (1968). A criterion for ductile fracture by the growth of holes. J. Appl. Mech..

[14] Gurson A.L. (1977). Continuum theory of ductile rupture by void nucleation and growth: Part I—Yield criteria and flow rules for porous ductile media. J. Eng. Mater. Technol..

[15] Koplik J., Needleman A. (1988). Void growth and coalescence in porous plastic solids. Int. J. Solids Struct..

[16] Pardoen T., Hutchinson J. (2000). An extended model for void growth and coalescence. J. Mech. Phys. Solids.

[17] Potirniche G., Hearndon J., Horstemeyer M., Ling X. (2006). Lattice orientation effects on void growth and coalescence in fcc single crystals. Int. J. Plast..

[18] Le Roy G., Embury J., Edwards G., Ashby M. (1981). A model of ductile fracture based on the nucleation and growth of voids. Acta Metall..

[19] Lubarda V., Schneider M., Kalantar D., Remington B., Meyers M. (2004). Void growth by dislocation emission. Acta Mater..

[20] Cui Y., Chen Z. (2016). Material transport via the emission of shear loops during void growth: A molecular dynamics study. J. Appl. Phys..

[21] Rudd R.E., Belak J.F. (2002). Void nucleation and associated plasticity in dynamic fracture of polycrystalline copper: An atomistic simulation. Comput. Mater. Sci..

[22] Ashby M.F., Gelles S., Tanner L.E. (1969). The stress at which dislocations are generated at a particle-matrix interface. Philos. Mag..

[23] Ashby M. (1970). The deformation of plastically non-homogeneous materials. Philos. Mag. A J. Theor. Exp. Appl. Phys..

[24] Bringa E.M., Cazamias J.U., Erhart P., Stölken J., Tanushev N., Wirth B.D., Rudd R.E., Caturla M.J. (2004). Atomistic shock Hugoniot simulation of single-crystal copper. J. Appl. Phys..

[25] Erhart P., Bringa E.M., Kumar M., Albe K. (2005). Atomistic mechanism of shock-induced void collapse in nanoporous metals. Phys. Rev. B.

[26] Davila L., Erhart P., Bringa E., Meyers M., Lubarda V., Schneider M., Becker R., Kumar M. (2005). Atomistic modeling of shock-induced void collapse in copper. Appl. Phys. Lett..

[27] Cao B., Bringa E.M., Meyers M.A. (2007). Shock compression of monocrystalline copper: Atomistic simulations. Metall. Mater. Trans. A.

[28] Neogi A., Mitra N. (2014). On shock response of nano-void closed/open cell copper material: Non-equilibrium molecular dynamic simulations. J. Appl. Phys..

[29] Neogi A., He L., Abdolrahim N. (2019). Atomistic simulations of shock compression of single crystal and core-shell Cu@Ni nanoporous metals. J. Appl. Phys..

[30] Potirniche G., Horstemeyer M., Wagner G., Gullett P. (2006). A molecular dynamics study of void growth and coalescence in single crystal nickel. Int. J. Plast..

[31] Zhao K., Chen C., Shen Y., Lu T. (2009). Molecular dynamics study on the nano-void growth in face-centered cubic single crystal copper. Comput. Mater. Sci..

[32] Tang T., Kim S., Horstemeyer M. (2010). Molecular dynamics simulations of void growth and coalescence in single crystal magnesium. Acta Mater..

[33] Mi C., Buttry D.A., Sharma P., Kouris D.A. (2011). Atomistic insights into dislocation-based mechanisms of void growth and coalescence. J. Mech. Phys. Solids.

[34] Xu S., Hao Z., Su Y., Yu Y., Wan Q., Hu W. (2011). An analysis on nanovoid growth in body-centered cubic single crystalline vanadium. Comput. Mater. Sci..

[35] Tang F.L., Cai H.M., Bao H.W., Xue H.T., Lu W.J., Zhu L., Rui Z.Y. (2014). Molecular dynamics simulations of void growth in *γ*-TiAl single crystal. Comput. Mater. Sci..

[36] Tanguy D., Mareschal M., Lomdahl P.S., Germann T.C., Holian B.L., Ravelo R. (2003). Dislocation nucleation induced by a shock wave in a perfect crystal: Molecular dynamics simulations and elastic calculations. Phys. Rev. B.

[37] Ruestes C.J., Bringa E.M., Stukowski A., Nieva J.R., Bertolino G., Tang Y., Meyers M. (2013). Atomistic simulation of the mechanical response of a nanoporous body-centered cubic metal. Scr. Mater..

[38] Xu X.T., Tang F.L., Xue H.T., Yu W.Y., Zhu L., Rui Z.Y. (2015). Molecular dynamics simulations of void shrinkage in *γ*-TiAl single crystal. Comput. Mater. Sci..

[39] Zhang Y., Jiang S., Zhu X., Zhao Y. (2017). Influence of void density on dislocation mechanisms of void shrinkage in nickel single crystal based on molecular dynamics simulation. Phys. E Low-Dimens. Syst. Nanostruct..

[40] Tang Y., Bringa E.M., Remington B.A., Meyers M.A. (2011). Growth and collapse of nanovoids in tantalum monocrystals. Acta Mater..

[41] Guan Y.L., Shao J.L., Song W. (2019). Molecular dynamics study on nanoscale void collapse in single crystal aluminum under 1D and 3D compressions. Comput. Mater. Sci..

[42] Xu Q., Li W., Zhou J., Yin Y., Nan H., Feng X. (2020). Molecular dynamics study on void collapse in single crystal hcp-Ti under hydrostatic compression. Comput. Mater. Sci..

[43] Epishin A.I., Svetlov I.L. (2016). Evolution of pore morphology in single-crystals of nickel-base superalloys. Inorg. Mater. Appl. Res..

[44] Plimpton S. (1995). Fast parallel algorithms for short-range molecular dynamics. J. Comput. Phys..

[45] Mishin Y. (2004). Atomistic modeling of the *γ* and *γ*-phases of the Ni–Al system. Acta Mater..

[46] Suzuki A., Mishin Y. (2005). Atomic mechanisms of grain boundary diffusion: Low versus high temperatures. J. Mater. Sci..

[47] Gornostyrev Y.N., Kontsevoi O.Y., Khromov K.Y., Katsnelson M., Freeman A.J. (2007). The role of thermal expansion and composition changes in the temperature dependence of the lattice misfit in two-phase *γ*/*γ* superalloys. Scr. Mater..

[48] Faken D., Jónsson H. (1994). Systematic analysis of local atomic structure combined with 3D computer graphics. Comput. Mater. Sci..

[49] Stukowski A. (2009). Visualization and analysis of atomistic simulation data with OVITO–the Open Visualization Tool. Model. Simul. Mater. Sci. Eng..

[50] Stukowski A., Bulatov V.V., Arsenlis A. (2012). Automated identification and indexing of dislocations in crystal interfaces. Model. Simul. Mater. Sci. Eng..

[51] Neogi A., Mitra N. (2017). A metastable phase of shocked bulk single crystal copper: An atomistic simulation study. Sci. Rep..

[52] Neogi A., Alam M., Hartmaier A., Janisch R. (2020). Anisotropic failure behavior of ordered intermetallic TiAl alloys under pure mode-I loading. Model. Simul. Mater. Sci. Eng..

[53] Neogi A., Mitra N. (2017). Shock induced deformation response of single crystal copper: Effect of crystallographic orientation. Comput. Mater. Sci..

[54] Stukowski A. (2014). Computational analysis methods in atomistic modeling of crystals. Jom.

[55] Hirth J.P., Lothe J., Mura T. (1983). Theory of Dislocations. J. Appl. Mech..

[56] Schäublin R., Yao Z., Baluc N., Victoria M. (2005). Irradiation-induced stacking fault tetrahedra in fcc metals. Philos. Mag..

[57] Neogi A., Mitra N. (2017). Evolution of dislocation mechanisms in single-crystal Cu under shock loading in different directions. Model. Simul. Mater. Sci. Eng..

